# The Fast Approval and Slow Rollout of Sputnik V: Why Is Russia’s Vaccine Rollout Slower than That of Other Nations?

**DOI:** 10.3390/epidemiologia2030027

**Published:** 2021-08-17

**Authors:** Elza Mikule, Tuuli Reissaar, Jennifer Villers, Alain Simplice Takoupo Penka, Alexander Temerev, Liudmila Rozanova

**Affiliations:** 1 Global Studies Institute, University of Geneva, 1205 Geneva, Switzerland; Tuuli.Reissaar@etu.unige.ch (T.R.); jennifer.villers@etu.unige.ch (J.V.); Alain.Takoupo@etu.unige.ch (A.S.T.P.); 2 Institute of Global Health, University of Geneva, 1202 Geneva, Switzerland; Alexander.Temerev@unige.ch (A.T.); Liudmila.Rozanova@unige.ch (L.R.)

**Keywords:** Russia, vaccination, SARS-CoV-2, Sputnik V, vaccine rollout, vaccine diplomacy, misinformation, vaccine hesitancy, EpiVacCorona, CoviVac

## Abstract

The emergence of the SARS-CoV-2 pandemic in the beginning of 2020 led to the deployment of enormous amounts of resources by different countries for vaccine development, and the Russian Federation was the first country in the world to approve a COVID-19 vaccine on 11 August 2020. In our research we sought to crystallize why the rollout of Sputnik V has been relatively slow considering that it was the first COVID-19 vaccine approved in the world. We looked at production capacity, at the number of vaccine doses domestically administered and internationally exported, and at vaccine hesitancy levels. By 6 May 2021, more first doses of Sputnik V had been administered abroad than domestically, suggesting that limited production capacity was unlikely to be the main reason behind the slow rollout. What remains unclear, however, is why Russia prioritized vaccine exportation. We provide three hypotheses that may contribute to explaining the slow domestic rollout: a generalized vaccine distrust among the Russian population, a desire to help less technologically advanced nations, and possible geopolitical incentives.

## 1. Introduction

On 11 August 2020, the president of the Russian Federation, Vladimir Putin, announced that Russia was the first country to approve a vaccine for SARS-CoV-2—the virus which had been responsible for the devastating state of the pandemic, death, adverse economic effects as well as psychological and social disturbances for months on end [1] This announcement, which was made exactly five months after the declaration of a pandemic by the WHO, provoked different reactions among the scientific community. While some welcomed this new development and saw it as a promising remedy for the adverse health and economic effects of the COVID-19 pandemic, others were sceptical about the fast approval of and lack of data transparency concerning this vaccine. Many academics questioned its safety and efficacy and pointed out that using a vaccine before the end of trials was unethical [1,2]. The Sputnik V vaccine was approved before the end of phase III clinical trials and only 55 days after the beginning of clinical trials, which in itself is unusual and raised concerns about its safety [2]. This could also be a possible explanatory pathway for why an initial triumph has garnered only questionable support at home, with only 34% of the Russian population willing to receive Sputnik V according to a poll carried out by the Levada centre [3]. Whether it was the rushed approval of Sputnik V or its political connotations that contributed to domestic vaccine hesitancy is not entirely clear. What is apparent, however, is that vaccine rollout in Russia has been mired in controversy both at home and abroad.

Despite the fact that Russia was the first country in the world to approve a vaccine against SARS-CoV-2, the domestic rollout has been relatively slow compared to other nations, with only 23% of the population vaccinated with one dose and 15% with both by 21 July 2021 [4]. The Russian government seems to be facing problems that could be linked to either vaccine hesitancy and disinformation, or production capacity, distribution, and logistics, or a combination of these. On 22 March, President Vladimir Putin confirmed that Russia had signed international sales deals for Sputnik V doses for 700 million people [5]. The extremely high export deal numbers contrast sharply with the low domestic rollout numbers. The European Commission’s President, Ursula von der Leyen, has emphasized this problem by saying that “We still wonder why Russia is offering, theoretically, millions and millions of doses while not sufficiently progressing in vaccinating its own people” [6]. Meanwhile the Deputy Chairman of the Security Council in Russia claimed the opposite when commenting on the foreign vaccine purchases by saying that ”As for foreign vaccines, nobody offers them to us especially, because every country wants to first of all satisfy its needs, it wants to vaccinate its population. This is absolutely normal, and our position is the same” [7] (author’s translation). These conflicting positions are central to our research as with our analysis we seek to crystallize the possible strategies regarding the distribution of Sputnik V in Russia and abroad as well as to summarize and compare the information regarding the vaccination campaign in Russia and the export deals for Sputnik V between Russia and other countries. It is also important to mention that the Russian people seem to be completely dependent on the vaccines produced in Russia because of the “third one is left out’’ (author’s translation, original—третий лишний) law, which implies that as long as two products (in this case—vaccines) of the same kind are produced in Russia, the government cannot purchase a foreign product of the same kind [8]. At this moment, four Russian COVID-19 vaccines are approved as safe and effective by the government of Russia. These are Sputnik V (inactivated adenovirus type 5 and 26 vector-based vaccine; Gamaleya institute), EpiVacCorona (SARS-CoV-2 synthetic Peptide-based vaccine; Vector State Research Center of Virology and Biotechnology), CoviVac (Inactivated SARS-CoV-2 vaccine; Chumakov Center), and Sputnik Light (a single dose version of Sputnik V containing only the inactivated adenovirus type 26 vector-based vaccine) [9,10,11]. It can be hypothesized that the unusually quick approval of Sputnik V and EpiVacCorona could be linked to the “third one is left out’’ law in order to avoid imports of foreign vaccines once they are approved. Although approved by the government of Russia, two of these vaccines—EpiVacCorona and CoviVac—are still undergoing clinical trials and, hence, still raise questions regarding safety and efficacy.

In this paper, we try to explain the reasons behind the slow domestic rollout of Sputnik V by looking at factors such as production and distribution, exportation, and vaccine hesitancy.

## 2. Materials and Methods

Our research combines methods of comparative statistics and an extensive literature review in order to crystallize the ongoing situation regarding vaccine production, availability, hesitancy, exports, and other factors linked to the COVID-19 vaccination campaign in the Russian Federation. In the analysis section, we identify and compare numbers regarding production rates, vaccination rates in Russia and export deals. We continue by exploring the importance of Sputnik V at the international level and the causes and burden of vaccine hesitancy at the national level. In the discussion, we summarize our findings and provide possible explanations for our observations and perspectives for further research. Our data sources are mainly based on Western and Russian media. Most numeric data have been gathered from Statista, Our World in Data, and several Russian sources (gogov.ru, стoпкoрoнавирус.рф). In addition to media sources, we also looked at several scientific publications and official Russian government statements including those published by the Ministry of Health (Minzdrav, Moscow, Russian Federation). The literature review was mainly conducted in English and Russian, and all translations in this paper from Russian to English were carried out by the authors.

## 3. Results

### 3.1. The COVID-19 Epidemic in Russia

**Figure 1:**
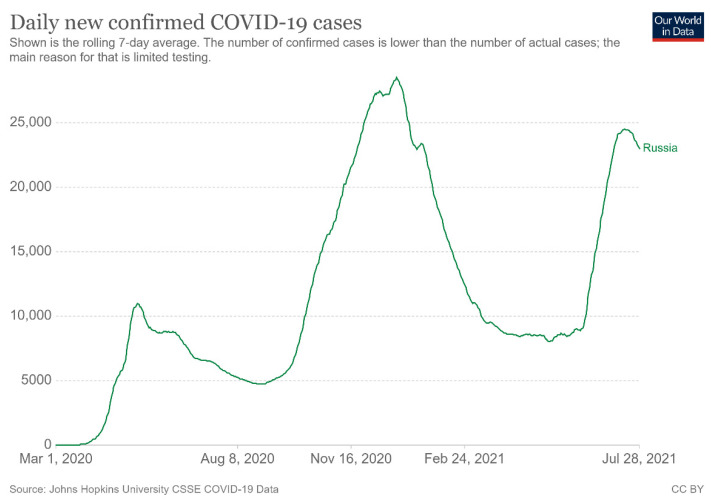
Daily number of confirmed COVID-19 cases in Russia (source: Our World in Data [16]).

The massive spread of COVID-19 in Russia is estimated to have started in March 2020, reaching a first peak on 11 May 2020, a second one on 24 December 2020, and a third one on 15 June 2021 (Figure 1). Official figures in Russia indicate, as of 28 July 2021, a cumulative number of COVID-19 cases greater than 6 million (~4.16% of the population) [12]. In response to the first wave, the government imposed a series of measures including a national lockdown, a stay-at-home order for residents aged 65+ years and for those suffering from chronic diseases, the obligation to wear a facemask in public transport and enclosed public spaces, the banning of mass events and public gatherings, and the interdiction for foreign nationals to travel to Russia for tourism purposes [13]. The lockdown was lifted on 11 May 2020, while other measures remained in place [14]. The maximum daily number of new cases reported during the second and third peaks were between 2 and 2.6 times higher than during the first peak (Figure 1). This could be explained by a combination of factors including higher testing rates [15] and the decision of the government not to implement a second lockdown.

Official COVID-19 mortality figures, as of 28 July 2021, indicate a death toll of almost 157,000 [12]—i.e., 0.1% of the population—suggesting lower mortality rates compared to Western nations (0.185% and 0.191% for the USA and the UK, respectively [17]). However, that number only accounts for deaths for which COVID-19 has been confirmed to be the primary cause of death by autopsy [18]. This way of tallying goes against the recommendations of the WHO to count all deaths “resulting from a clinically compatible illness, in a probable or confirmed COVID-19 case, unless there is a clear alternative cause of death that cannot be related to COVID disease (e.g., trauma)” [19]. Data published by Russia’s federal statistics service Rosstat present the following figures for the year 2020: 57,019 official COVID-19 deaths, 162,429 COVID-19-related fatalities, and 323,000 excess deaths compared with the year 2019 [18]. The number of COVID-19-related fatalities is 2.8 times higher than the official count of COVID-19 deaths, suggesting a significant under-reporting on behalf of the Russian authorities. According to a tracker from The Economist, which counts excess mortality since the country’s first 50 COVID-19 deaths, Russia ranks 5th worldwide in excess mortality per population size [20]. As a comparison, the USA and the UK rank 26th and 36th, respectively.

### 3.2. Vaccine Production and Distribution

**Figure 2:**
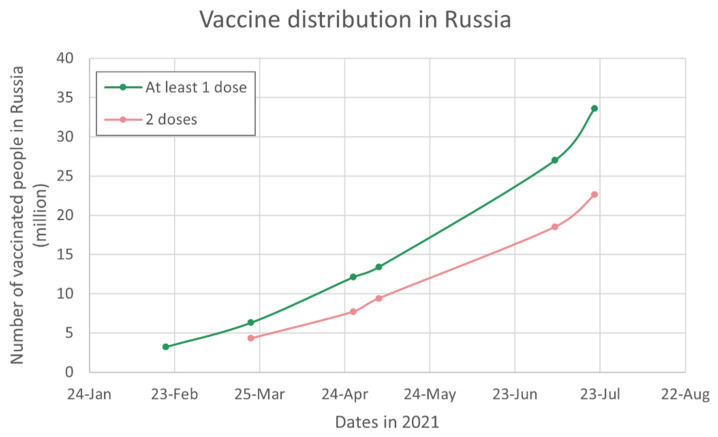
Number of vaccinated people in Russia as a function of time (data collected from official communications from the Russian government [4,5,30,31,32]).

While Russia was the first country to approve a COVID-19 vaccine without limiting conditions—Sputnik V on 11 August 2020—the vaccine rollout in Russia has been surprisingly slow compared to that of other nations. Mass vaccination in Russia was launched on 18 January 2021 with the ambitious target to vaccinate 68.6 million people (~60% of the adult population) by June 2021 [21,22]. In a similarly ambitious statement, Kirill Dmitriev, head of the Russian Direct Investment Fund (RDIF), said that they “will be able to provide about 700 million people with the [Sputnik V] vaccine this year” [23] (author’s translation). In this section, we examine the number of vaccine doses produced in Russia as well as the number of vaccine doses distributed within Russia and abroad. In 2020, only 2 million kits (1 kit = 2 doses) of Sputnik V were produced. One reason for this low level of production was a problem with the manufacturing of the second component of the two-shot vaccine [24]. Since the beginning of 2021, production has steadily increased up to 11 million kits during the first quarter of 2021 and 20 million during the second quarter [25]. While releasing 32 million kits of vaccine by the end of June was a significant progress, it fell short of the predictions made by the Kremlin during the first quarter (see Table 1). Similarly, vaccination rates remained quite low during the first half of the year, with only 18.5 million Russians having been fully vaccinated by July 7 (see Figure 2), which is 3.7 times lower than the 68.6 million required to reach the 60% herd immunity target.

Interestingly enough, both production and vaccination rates have risen steeply in July. The number of vaccine kits released in 2021 reached 43.8 million on 16 July [25] while the number of individuals fully vaccinated reached 22.6 million on 21 July (see Figure 2). Russia is currently vaccinating an average of 288,000 people per day (https://gogov.ru/articles/covid-v-stats, accessed on 31 July 2021) [29], which is twice the number of people that were vaccinated per day in June (https://gogov.ru/articles/covid-v-stats, accessed on 10 June 2021). [29]. Maintaining the current pace, Russia can expect to reach the 60% threshold within 113 days [29]. However, with the emergence of more contagious variants, the government has raised the herd immunity target to 80% [4].

Whether vaccination rates have increased as a result of increased production or thanks to other factors remains unclear. As of May 6, more people outside of Russia (over 20 million [33]) had received their first dose of Sputnik V when compared to Russian citizens (13.4 million [31]), which suggests that delays in production are unlikely to be the main reason for the slow vaccine rollout in Russia. Other possible factors will be discussed in the next sections of this paper.

### 3.3. Use of Russian Vaccines Abroad

Sputnik V was the first COVID-19 vaccine to be authorised for mass vaccination. However, its safety and efficacy remained controversial up until the publication of phase III data in the Lancet that confirmed a 91.6% efficacy against SARS-CoV-2 [34]. By now, 68 countries have approved the use of Sputnik V [35] with over 50 countries having placed orders for it [36]. As of 29 July, export deals have been sealed for over 500 million doses of Sputnik V, with India purchasing the highest quantity (Table 2). While the number of doses ordered is impressive, many countries only received a small fraction of what they have ordered (Table 2). Production as well as logistical challenges including cold storage requirements and the lack of direct flights from Russia have contributed to the delay of Sputnik V exportation [37]. While some of these challenges were solved through the production of freeze-dried doses of Sputnik V that can be stored at normal refrigerator temperatures [38,39], one major challenge remained: balancing export deals and domestic needs given lower-than-expected production capacities. Foreign production of Sputnik V is another strategy developed by Russia to cover foreign demands and avoid shortages at home. About 3 billion doses of Sputnik V are planned to be produced abroad in more than ten different countries so far with the highest quantities to be produced in the Republic of Korea and India, i.e., 1.85 and 1.15 billion doses, respectively (Table 3). However, very few countries have actually started to mass-produce the vaccine, and most are still relying on Russian-made doses (Table 3).

While international requests for Sputnik V have been increasing in the past months, the approval process by some countries and medical agencies has been significantly slower. For instance, the European Medicines Agency (EMA) has conducted a Good Clinical Practice inspection—a review process that checks the “international ethical and scientific quality standards for designing, recording, and reporting trials” [89]. None of the other vaccines approved in Europe have been through such an investigation. It is, however, a standard procedure that “may be triggered by issues arising during the assessment of the dossier or by other information such as previous inspection experience” [90]. Among possible triggers, we found that a group of 10 scientists, including Russian scientist Vasiliy Vlassov from the Higher School of Economics in Moscow, sent a letter to the EMA highlighting an “unexpected homogeneity of vaccine efficacy between age groups” [91], which could be an indicator of data manipulation. These concerns were recently published in the Lancet [92]. Two member states of the EU—Hungary and Slovakia—have decided not to wait for the EMA review to make an emergency approval of Sputnik V and to order 2 million doses each from Russia (Table 2). However, Slovakia’s approval process was everything but smooth. In April, Slovakia’s State Institute for Drug Control claimed the Sputnik V vaccines delivered to the country differed from those reviewed by international scientists and by the European Union regulator. The institute said it had also not received enough information about Sputnik V to be able to assess its benefits and risks [93]. The batches were later sent to Hungary for reanalysis in an EU-certified laboratory and were found to be satisfactory, which led to Slovakia’s approval of Sputnik V on 26 May [94,95]. In a similar vein, on 26 April, the Brazilian national drug regulator ANVISA rejected the Sputnik V vaccine on the grounds that the batches they analyzed showed adenoviruses with breeding capacity in the vaccine compound, which could present a safety risk for people with low immunity and chronic respiratory diseases [96]. While the concerns with replicating viruses have not been fully resolved, new documents provided to ANVISA suggest a substantially lower and more acceptable amount. On 5 June, ANVISA decided to approve the use of Sputnik V with restrictions. The vaccine will only be provided to healthy people in places where it is possible to monitor and treat adverse reactions and to no more than 1% of the population in 6 states to allow for strict monitoring [97]. These recent developments could be expected to further reinforce the high levels of vaccine hesitancy both in Russia and abroad.

### 3.4. Vaccine Hesitancy

**Figure 3:**
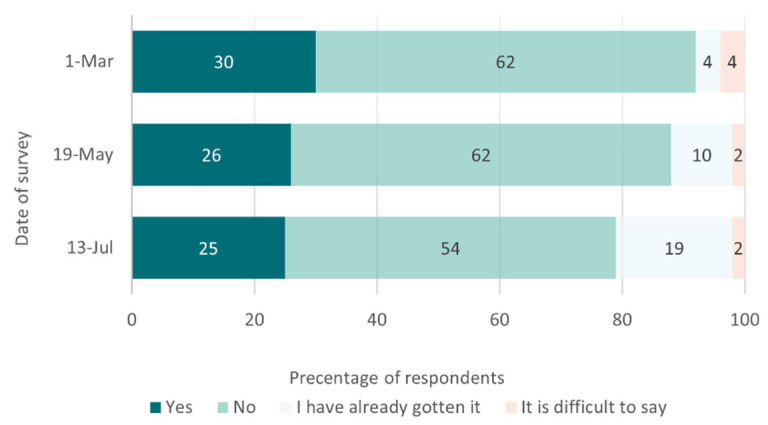
Willingness to receive the COVID-19 vaccine Sputnik V in Russia, evolution over time from March to July 2021 (data collected from three Levada surveys) [3,100,101].

While vaccine shortages have been reported in most regions, large cities such as Moscow were found to have an oversupply of vaccine doses—exceeding the number of people wishing to be vaccinated—according to a survey of medical doctors conducted by the Levada center at the end of March [98]. On 18 May, officials reported that 1.4 million residents of Moscow had received at least one dose of COVID vaccine [99]. This figure represents only 11% of the total population of Moscow. Taken together, these data suggest that the majority of Muscovites do not wish to take the vaccine. This observation is consistent with the results of two other Levada surveys which found in February and April that 62% of respondents were not willing to be vaccinated with Sputnik [100,101]. Only recently, the share of people who are not ready to be vaccinated has started to drop—from 62% in April to 54% in June (Figure 3).

Vaccination is known to be one of the best methods for infectious disease prevention. However, the rise in vaccine hesitancy threatens to jeopardize public health efforts and has been listed as one of the top 10 threats to global health by the World Health Organization back in 2019 [102]. Today, with the COVID-19 pandemic, the threat of vaccine hesitancy has become more evident. In a global health survey published in Nature Medicine that looked at potential COVID-19 vaccine acceptance rates in 19 countries, respondents from China gave the highest proportion (89%) of positive responses when asked if they would accept a “proven, safe, and effective vaccine”, while respondents in Russia gave the lowest number (55%) of positive responses [103]. Such a high level of vaccine hesitancy may prevent Russia from reaching herd immunity, despite the fact that 4 vaccines have already been fully approved for domestic vaccination. Possible reasons that have been raised to explain this level of vaccine hesitancy include a negative attitude of the society towards vaccination in general, a distrust of existing COVID vaccines, the absence of fear—57% of Russians are not afraid of contracting the coronavirus [3]—and the misbelief that once you have been infected you do not need the vaccine anymore [98,104]. Among medical doctors, the level of confidence in Sputnik V was found to be higher with 69% of them trusting the safety and efficacy of the vaccine. The level of confidence dropped to 48% and 41%, respectively, when it came to CoviVac and EpiVacCorona [98]. The lower level of confidence for the two newest vaccines was, as expected, mainly explained by the lack of information regarding their safety and efficacy. While Sputnik V has passed phase III trials and was found to be 91.6% effective [34], there is no data on the effectiveness of EpiVacCorona and CoviVac as these are still undergoing clinical trials. Hence, one could question whether Russia’s rushed approval of vaccines—before the completion of phase III trials—is not partly responsible for the low level of confidence Russians have placed in the aforementioned vaccines. A common reason cited by people who do not wish to be vaccinated is the fear of side effects. The only side effects that have been reported so far after the injection of Sputnik V are flu-like illness, injection site reactions, headache, and weakness/lack of energy [34]. These side effects are common to most vaccines, including inactivated adenovirus vaccines such as Sputnik V, AstraZeneca, and Johnson & Johnson [105]. Another rare side effect of blood clotting has been noticed in AstraZeneca and Johnson & Johnson vaccines. German scientists confirmed this blood clotting side effect with AstraZeneca vaccine and identified a plausible molecular mechanism [106]. They also emphasized that this side effect could be characteristic to all vector-based vaccines involving adenoviruses [107]. However, so far, no blood clotting side effects have been reported with the Sputnik V vaccine. Possible explanations range from under-reporting of side effects to a superior vaccine purification technology [108]. However, data from Argentina and San Marino on more than 2.8 million doses of Sputnik V strongly suggest that the vaccine is safe and effective decreasing the likelihood of an under-reporting problem [109].

## 4. Discussion

While the official death count suggests that Russia was only mildly affected by the pandemic, the number of COVID-19-related fatalities as well as the excess mortality—both published by Russia’s federal statistics service Rosstat—paint a different picture. Russia ranks 5th worldwide in excess mortality per population size, suggesting widespread disruptions in the functioning of the health system. The peak of the second wave in Russia was recorded on 24 December 2020, less than one month before the launch of Russia’s mass vaccination campaign. Given that Russia was the first country to approve a COVID-19 vaccine and given its worrying epidemiological situation, one would have expected the vaccination rates to quickly rise as they did in the UK—another country that has developed and produced its own vaccine. However, vaccination rates in Russia have remained surprisingly low, with only 15.5% of the Russian population having received their first dose by the end of June (as opposed to 66% in the UK and 54% in the USA [110]). In this paper, we used official figures and public surveys to identify the factors that might explain this slow vaccine rollout. First, we looked at the number of doses produced, the number of doses administered within Russia, and the number of doses sent for exportation. While production rates have been lower than expected—with only 32 million kits of two doses released into civilian circulation by 25 June 2020—our analysis suggests that low production rates alone cannot explain the slow domestic rollout. Delays in vaccine production have occurred with all commercially available COVID vaccines. However, countries’ reactions to those delays have differed. Most Western nations have responded to such shortages by vaccine hoarding strategies. Conversely, Russia has exported the majority of its production (at least until 6 May 2021). We identify three hypotheses that could explain Russia’s choice to favor exports: (1) the high levels of vaccine hesitancy in Russia, (2) the conviction that a fair and equitable distribution of vaccines throughout the world is necessary if we want to put an end to the pandemic, and (3) possible geopolitical gains that could arise from sealing export deals with LMICs. Each of these hypotheses—and possible combinations of them—will be discussed in the next paragraphs.

Firstly, the slow vaccine rollout could be explained in terms of low domestic demand for COVID-19 vaccines [104]. This, in turn, has linkages to vaccine hesitancy which has recently been increasingly linked to the spread of mis- and disinformation in various countries [111,112]. Now more than ever, the abundance of information—digital overload one might call it—is fuelling an infodemic whereby both misinformation (false information which was created or spread unintentionally) as well as disinformation are spreading like wildfire. People flock increasingly to online sites and social media in search of information and guidance. The Reuters Institute for the Study of Journalism has reported that online news consumption rose considerably as quarantines began globally [113]. While it is important to have access to scientifically evidenced, factual and balanced information about the COVID-19 outbreak, the unprecedented spread of fake news has demonstrated the power of alternative media in undermining the authority of political institutions and it could be argued that mistrust in traditional media and the search for alternative sources of information could be a driving force for vaccine hesitancy in Russia and globally. In accordance with this hypothesis, the threat of an infodemic and the “nihilism of citizens” were cited by members and spokespersons of the government to explain the slow vaccine rollout in Russia [114,115]. On 16 June—during the peak of the third wave—Moscow became the first region to introduce mandatory vaccination for some categories of workers [116]. It was soon followed by many regions of Russia, with Madagan becoming on July 26 the 40th region of Russia to introduce compulsory vaccination against SARS-CoV-2 [117]. Vaccination rates have risen steeply in the second half of July, suggesting that vaccine hesitancy was indeed one of the main drivers for the slow domestic rollout.

A second factor that may have influenced the rollout and uptake of domestic vaccines is an overall issue of vaccine shortages in Russia, which may have been precipitated by foreign demand and numerous export deals [104]. In times of COVID-19 where national vaccine rollouts have highlighted the pervasive inequality and the tendency for vaccine nationalism, LMICs in general and Latin American countries in particular have found themselves in a position that is akin to being between a rock and a hard place. Opting for vaccines that had not completed phase III of clinical trials over waiting for wealthy countries to stop stockpiling was predominantly the choice made by lower-income countries. Instead of hoarding vaccine doses, Russia started providing LMICs with vaccines long before reaching any form of local herd immunity. This is in agreement with the motto of the WHO General Director Tedros Adhanom Ghebreyesus “no one is safe until everyone is safe” [118] and has been expressed in similar terms by Kremlin spokesman Dmitry Peskov “we believe that there should be as many doses of vaccines as possible so that all countries, including the poorest, have the opportunity to stop the pandemic” [119]. This narrative of helping LMICs to address the heavy toll of COVID-19 is on the face of it laudable and compatible with the utopian idea of equal vaccine distribution on the global level, but critics increasingly point to Sputnik V being used as a geopolitical soft power weapon [2,120].

Lastly, the slow rollout of vaccines in Russia and the numerous export deals that contrast with limited production capacities could also be explained by geopolitical interests, nation branding and the exercise of soft power. According to the American political scientist Joseph Nye, “Soft power is the ability to obtain preferred outcomes by attraction rather than coercion or payment” [121]. It refers to the ability to shape preferences of different groups of people by offering them what they might find appealing. Following Nye’s theory, we could hypothesize that the high numbers of Sputnik V vaccine exports to mainly LMICs (notably, those that have had good diplomatic relations with Russia in the past) could be considered as an exercise of soft power, since Russia would be providing these countries with very attractive goods (COVID-19 vaccines) as well as the “return to normalcy” and probably would in return expect some willingness to cooperate in matters of foreign politics. COVID-19 vaccines are currently not only a public health instrument, but since the economy and social well-being of a country is highly dependent on the improvement of the sanitary situation, they also serve as a powerful political instrument, making them one of the most demanded technologies in the world [122]. It would be difficult to find a product that was more appealing and wanted by governments all around the world at the moment, which in turn makes the COVID-19 vaccine the perfect tool to exercise soft power. Therefore, it could be hypothesized that the Russian government has prioritized exportation in order to increase international geopolitical influence by exercising soft power and branding Russia as the “savior” [120]. We consider this as an interesting and a highly interdisciplinary perspective for further research.

Since vaccination affects and is in turn influenced by many social and political systems, we believe that an interdisciplinary and systems thinking approach would be beneficial to better understand domestic and international vaccine rollouts. Vaccine coverage cannot be explained solely by production and infrastructure capacities and we believe that a Global Health approach which would include geopolitical and sociocultural analyses should be incorporated in studies that look at worldwide biotechnology distribution in the context of emerging infectious diseases. An interesting perspective for further research would be to carry out a cost/benefit analysis regarding the choice to prioritize vaccine exports over reaching herd immunity. Finally, it would be interesting to study the root causes of vaccine hesitancy in order to understand why some populations are more receptive to misinformation and how that issue could be addressed in the future.

This study presents a certain number of limitations. One major limitation comes from the difficulty to collect accurate data. The numbers presented by Russian officials are often scarce and are not always the most representative of reality—as illustrated by the wide gap between the official COVID-19 death toll and excess mortality. Another limitation of this study is that we had to rely heavily on reports from the media that sometimes present biased views depending on their geopolitical orientation. Finally, none of the authors were living in Russia during the COVID-19 pandemic. On the other hand, some of the authors were proficient in Russian and as such were able to also gather information from the Russian media and official government sources.

## 5. Conclusions

While Russia was the first country to approve a COVID-19 vaccine, its domestic vaccine rollout has been slow compared with that of other nations. Russia has only been able to fully vaccinate 22.6 million of individuals so far, which represents one third of their 60% herd immunity target (which in itself has been recognized to be insufficient to counter the more contagious delta variant [4]). Production shortages have likely played a role in the slow rollout of Sputnik V in Russia and abroad. However, the comparison between the number of doses produced and exported suggests that Russia has favored exportation. What is not unequivocally clear, however, is the reason behind that choice. Possible explanatory pathways range from the belief that vaccines should be accessible to all countries of the world—including less technologically advanced ones—to the inability to address domestic issues of vaccine hesitancy, to the strategic politicization of vaccines in an effort to increase Russia’s influence in LMICs and to boost Russia’s image as a technological power and world savior. All of these constitute interesting pathways of further research. As previously mentioned, it is highly important to carry out interdisciplinary studies when it comes to complex phenomena in global health, such as vaccine rollouts during a pandemic. Finally, we conclude by stressing that the politicization and instrumentalization of health-related technologies should be avoided at all costs as it is harmful for the promotion of global health. Health is too important to be politicized.

## Figures and Tables

**Table 1:** Comparison between the vaccine production targets for the end of June 2021 and the actual number of kits produced by the end of June 2021.

	Number of Kits Planned	Number of Kits Released [26]
Sputnik V	83 M [27]	~30 M
EpiVacCorona	5.4 M [27]	1.5 M
CoviVac	775 K [5]	352 K
Total	89 M [28]	32 M

**Table 2:** Number of doses of Sputnik V ordered and received from Russia as of 31 July 2021.

Country	Ordered from Russia (Million) [36]	Received from Russia	As of (Date), 2021	References
India	250	3 M of 1st dose 0.36 M of 2nd dose	20 June	[37]
Iran	60	2 M	29 July	[40]
Turkey	50	0.4 M	14 June	[41,42]
Egypt	25	0.21 M	24 June	[43]
Mexico	24	4.1 M	29 July	[40]
Argentina	22	9.375 M of 1st dose 2.493 M of 2nd dose	15 July	[44]
Vietnam	20	-	-	[45]
Angola	12	0.04 M	May	[40]
Brazil	10	First batch of 1.145 M	Expected on 28 July	[46]
Venezuela	10	1.43 M (but not enough 2nd doses)	16 June	[47]
Guatemala	8	0.15 M	End of June	[40]
Nepal	8	-	-	[48]
Kazakhstan	6	0.022 M (now producing its own stock)	13 April	[49]
Bolivia	5.2	0.745 M	End of May	[40]
Honduras	4.2	0.046 M of 1st dose	30 April (2nd doses still missing by 30 July)	[50]
Ghana	3.4	0.02 M	Early July	[40]
Palestine	2	0.01 M + 0.06 M donated by UAE + 0.02 M donated by Chechenia	19 July	[51,52,53]
Hungary	2	2 M	4 June	[54]
Serbia	2	0.4 M (now producing its own stock)	23 March	[55]
Slovakia	2	0.2 M (0.16 M sold back)	1 March	[56]
Paraguay	1.4	0.38 M of 1st dose	20 July (2nd doses expected on 18 August)	[57]
Uzbekistan	1	0.23 M of 1st dose 0.14 M of 2nd dose	15 July	[58]
Algeria	1	0.05 M	8 April (expecting 0.7 M in June)	[59,60]
Tunisia	0.5	0.2 M	29 July	[61]
Kyrgyzstan	0.5	0.12 M	17 June	[62]
Bosnia & Herzegovina	0.4	0.04 M	17 March	[63]
Azerbaijan	0.3	0.08 M	10 June	[62]
North Macedonia	0.2	0.048 M donated by Serbia	28 April	[64]
Belarus	0.17	Now producing its own stock		[65]
Armenia	-	0.105 M	9 July	[62]
United Arab Emirates	-	>0.081 M	8 June	[66]
Mongolia	-	0.08 M	2 July	[67]
Philippines	-	0.065 M	7 July (expecting 0.17 M)	[68]
San Marino	-	0.039 M	End of May	[69]
Montenegro	-	0.02 M donated by Serbia + 0.01 M	6 June (expecting 0.04 M)	[70,71]

**Table 3:** Number of doses of Sputnik V to be produced abroad and number of doses already produced abroad as of 31 July 2021.

Country	Planned Production for 2021 (Million) [36]	Doses Already Produced	Date	References
Republic of Korea	1850	-	Planned to start in August	[72]
India	852	-	Planned to start in September	[73]
China	260	-	-	[74]
Egypt	40	-	Planned to start in the fall	[75]
Argentina	12	1.3 M of 1st dose 1 M of 2nd dose	15 July	[44]
Italy	10	Launched production of 1st test batch	16 June	[76]
Brazil	8	1st batch (0.1 M)	20 May	[77]
Mexico	7 to 20 per month	1st test batch	8 July	[78,79]
Vietnam	5 per month	1st test batch (0.03 M)	21 July	[80]
Serbia	4	Tens of thousands	5 July	[81]
Algeria	2.5 per month	-	Planned to start in September	[82]
Kazakhstan	2	(*)		[83]
Belarus	0.5 per month	Mass production launched	25 March	[65,84]
Iran	-	1st test batch	26 June	[85]
Armenia	-	Has started production	1 July	[86]
Uzbekistan	-	-	Planned to start in June	[62]
Bahrain	-	-	-	[87]
Turkey	-	-	-	[88]

(*) By 29 July 2021, 5.27 million people have been vaccinated with the first component of the vaccine, and 3.53 million people in Kazakhstan have received both doses. Most of the country’s residents are vaccinated with the Russian Sputnik V vaccine, which is produced locally.

## Data Availability

Data is contained within the article.
